# Alpha-Fetoprotein (AFP) and AFP-L3 Is Most Useful in Detection of Recurrence of Hepatocellular Carcinoma in Patients after Tumor Ablation and with Low AFP Level

**DOI:** 10.3390/v14040775

**Published:** 2022-04-08

**Authors:** Madison Force, Grace Park, Divya Chalikonda, Christopher Roth, Micah Cohen, Dina Halegoua-DeMarzio, Hie-Won Hann

**Affiliations:** 1 Department of Medicine, Thomas Jefferson University Hospital, Philadelphia, PA 19107, USA; madison.force@jefferson.edu (M.F.); divya.chalikonda@jefferson.edu (D.C.); dina.halegoua-demarzio@jefferson.edu (D.H.-D.); 2 Department of Medicine, Division of Gastrotenterology and Hepatology, Thomas Jefferson University Hospital, Philadelphia, PA 19107, USA; grace.park@jefferson.edu; 3 Department of Radiology, Thomas Jefferson University Hospital, Philadelphia, PA 19107, USA; christopher.roth@jefferson.edu; 4 Department of Radiology, Einstein Medical Center, Philadelphia, PA 19141, USA; cohenmic@einstein.edu

**Keywords:** AFP, AFP-L3, hepatocellular carcinoma (HCC), hepatitis B virus (HBV), chronic hepatitis B (CHB)

## Abstract

Hepatocellular carcinoma (HCC) is the most common primary malignancy of the liver and is a leading cause of mortality worldwide. While there are many risk factors for HCC including alcohol, obesity, and diabetes, hepatitis B virus (HBV) and hepatitis C virus (HCV) infection still account for the majority of HCC worldwide. Globally, HBV is the leading risk factor for HCC. Patients with chronic hepatitis B (CHB) and advanced liver disease are at high risk for HCC. Screening for HCC is done routinely with ultrasound with or without alpha-fetoprotein (AFP) at six-month intervals. The combination of ultrasound and AFP has been shown to provide some additional detection of 6–8% of cases compared to ultrasound alone; however, this also increases false-positive results. This is because AFP can be elevated not only in the setting of HCC, but also in chronic hepatitis, liver cirrhosis, or ALT flare in CHB, which limits the specificity of AFP. AFP-L3 is a subfraction of AFP that is produced by malignant hepatocytes. The ratio of AFP-L3 to total AFP is reported as a percentage, and over 10% AFP-L3 is consistent with a diagnosis of HCC. Here, we review five cases of patients with CHB, cirrhosis, and HCC, and their levels of AFP and the AFP-L3% at various stages of disease including ALT flare, cirrhosis, initial diagnosis of HCC, and recurrence of HCC. These cases emphasize the utility of AFP-L3% in identifying early, new or recurrent HCC prior to the presence of imaging findings.

## 1. Introduction

Hepatocellular carcinoma (HCC) is the most common primary malignancy of the liver and a leading cause of mortality worldwide [1]. HCC accounts for 600,000 deaths worldwide (50% in China) and 10,000 deaths in the US annually. Globally, 53% of HCC is attributed to hepatitis B virus (HBV) and 25% to hepatitis C virus (HCV) [1,2,3]. The disease burden is highest in endemic regions of HBV infection [1].

There are many risk factors for HCC including alcohol, obesity, and diabetes, but HBV and HCV infections account for the majority of HCC worldwide. Globally, HBV is the leading risk factor for HCC [1].

For patients at high risk for HCC, surveillance by abdominal ultrasound (US) is used as the first line modality, followed by dynamic computed tomography (CT) or magnetic resonance imaging (MRI). Imaging studies should be performed every 6 months (or 3 months if indicated) for detection of HCC. Most guidelines recommend imaging with concomitant alpha-fetoprotein (AFP) level for HCC surveillance. However, AFP can be elevated not only in HCC, but also other conditions such as pregnancy, chronic liver inflammation, and during ALT flare. Therefore, AFP-L3 has been used as the specific tumor marker for HCC during the recent years but not as routinely as AFP alone [4].

## 2. History of AFP

AFP is an alpha1 globulin containing glycoprotein and its level is increased during pregnancy due to the production by the fetal liver. Elevated AFP level disappears from serum within 3 weeks after childbirth [5].

Furthermore, elevated AFP during acute liver injury indicates active regeneration of liver cells. Serum studies made before and after partial hepatectomy experiments in animals have indicated that hepatocyte proliferation is related to alpha-fetoprotein production [6]. In acute liver failure due to acetaminophen liver injury, an increase in AFP was strongly associated with a favorable outcome [7].

AFP as a marker for liver tumor was first demonstrated in mice by Abelev et al. in 1963 and in patients with liver cancer by Tatarinov in 1963 and 1964 [8,9,10]. Increased AFP was also found in yolk sac tumors as described by Abelev et al. and Masopust et al. [8,9,10,11].

## 3. AFP L1, L2, and L3

AFP is a glycosylated protein and based on its binding capability to lectin Lens Culinaris Agglutinin (LCA), and total AFP can be separated into three different glycoforms, AFP-L1, AFP-L2, and AFP-L3. AFP-L1 does not bind LCA, and it is the major glycoform produced by nonmalignant hepatocytes in patients with chronic hepatitis B (CHB) infection or cirrhosis [4,5,6]. AFP-L2 has intermediate binding affinity to LCA and is produced by yolk sac tumors and can be detected in the maternal serum during normal pregnancy [12]. AFP-L3 does bind LCA and is produced by malignant hepatocytes [4]. The current cut-off value for L3% is 10%. Patients with AFP-L3 > 10% are consistent with HCC and could be defined as more aggressive cancers, as HCC cells producing more AFP-L3 have been found to have a tendency for early vascular invasion and intra-hepatic metastasis [4].

Ultrasound has a sensitivity of 78% for detecting HCC at any stage and 45% for detecting early-stage HCC [13,14]. MRI and dynamic contrast-enhanced CT are both more sensitive for detecting HCC but are not recommended as the primary imaging modality for screening [15]. An alternative screening method to ultrasound alone is to include AFP at 6-month intervals with ultrasound. The sensitivity of ultrasound plus AFP for detecting HCC at any stage is 97% and 63% for detecting early-stage HCC [13,14].

The AFP-L3 can be detected in the serum of approximately 35% of the patients with small HCC (<2 cm). The AFP-L3-positive HCC has potential for rapid growth and early metastasis. Compared to imaging techniques, it has been shown to have 9–12 months of lead-time in early HCC recognition. Combined sensitivity of AFP-L3 for HCC is 56%, with a specificity of >95 [13,14].

## 4. Methods

In this study we included patients with CHB who were followed by a hepatologist and getting screened regularly for HCC between the years of 2008 and 2011. Selection of patients was based on the presence of laboratory data, corresponding stored serum samples, and imaging results. This case series study protocol was approved by the Institutional Review Board at Thomas Jefferson University Hospital.

AFP and AFP-L3% were measured in serum samples of patients collected at various times throughout their illness course. AFP, AFP-L3, and DCP were measured using a microchip capillary electrophoresis and liquid-phase binding assay on a µTASWako i30 auto analyzer (Wako Diagnostics, Wako Life Sciences, Inc., Mountain View, CA, USA) [16]. The measurement range for AFP was between 0.3 and 1000 ng/mL. Previously, AFP-L3% was only able to be calculated for AFP levels > 10 ng/mL. However, the auto analyzer used has high sensitivity for AFP-L3% at low AFP concentrations. AFP-L3% can be as long as the AFP is over 0.6 ng/mL [16].

Imaging studies including CT scans and MRIs were retrospectively reviewed after each of the patients were selected and select representative images were chosen by Dr. Christopher Roth to be included in the figures.

## 5. Case Series of Patients with Chronic HBV Infection and Retrospective Review of AFP-L3% throughout Disease Course

At our institution, AFP has been used routinely to screen for HCC in patients who are at risk for development of HCC. AFP with AFP-L3 is measured when there is a strong suspicion of HCC recurrence after tumor ablation, and in particular when a high level of AFP was noted in patients without risk for HCC. We measured total AFP and %L3 in the serial serum specimens collected over time, in patients with ALT flare, liver cirrhosis, HCC, and recurrent HCC and along with radiological examination. The patients presented here have been followed in our Liver Disease Prevention Center due to their diagnosis of HCC and CHB.

## 6. ALT Flare/Cirrhosis

### 6.1. Case 1: A 59-Year-Old Male during ALT Flare

Case 1 studies a 59-year-old male who was diagnosed with HBV infection during blood donation at age 20. At age 49, he presented with fatigue, ALT 625 U/L, HBeAg positive, HBV DNA 4.2 × 10^9^ copies/mL, and AFP 2148 ng/mL. Abdominal ultrasound showed a coarse liver echogenicity without tumor. He was started on entecavir, and 6 months later ALT was down to 149 IU/L and HBV DNA became undetectable. At this time his AFP was 1896 ng/mL.

As shown in Table 1, the patient’s serum specimen that showed AFP 1896 ng/mL was retrospectively tested for % of L3, which was 4.4%. This low value of AFP-L3% suggested that his elevated AFP was from regenerating liver cells during the ALT flare. One year and two years later his AFP levels were 3 ng/mL with % L3 AFP of 0.5 with normal ALT, suggesting resolution of the flare.

### 6.2. Case 2: A 57-Year-Old Male with Liver Cirrhosis

A 57-year-old male was infected with HBV perinatally. He presented with fatigue, abdominal distension, and lower extremity swelling, and an MRI done at that time showed cirrhosis and signs of portal hypertension but no liver masses. Labs demonstrated an ALT 331 IU/L, AST 578 IU/L, HBV DNA 7.47 × 10^8^ copies/mL, and AFP 558 ng/mL. He was started on tenofovir 300 mg daily. Three months after the initiation of treatment the ALT normalized, and 3 months later the HBV DNA became undetectable. Both ALT and AFP were elevated initially and decreased one year after the antiviral treatment with complete resolution of AFP to 6.5 ng/mL. Retrospective measurement of the serum samples for AFP and %AFP-L3 during the antiviral treatment showed nearly all below 10%, which is consistent with AFP elevation due to cirrhosis/ALT flare rather than HCC as shown in Table 2.

### 6.3. Case 3: A 59-Year-Old Male with HCC

A 59-year-old male with CHB was started on lamivudine 150 mg daily that resulted in a negative HBV DNA and HBeAg seroconversion in 5 months of therapy. Three months later, AFP was 544.5 ng/mL, and abdominal MRI showed a 3.3 × 2.4 cm enhancing mass compatible with HCC. He underwent laparoscopic radiofrequency tumor ablation with success. Four months later, the AFP was 2.2 ng/mL, and the MRI showed no recurrence or new lesions of HCC. Four months later, the AFP was 8.4 ng/mL, and the abdominal MRI showed two new small lesions, concerning for the recurrence of HCC. Retrospective evaluation of %L3 of this AFP sample showed 74.2% consistent with HCC (Table 3 below).

### 6.4. Case 4: A 50-Year-Old Male

**Figure 1:**
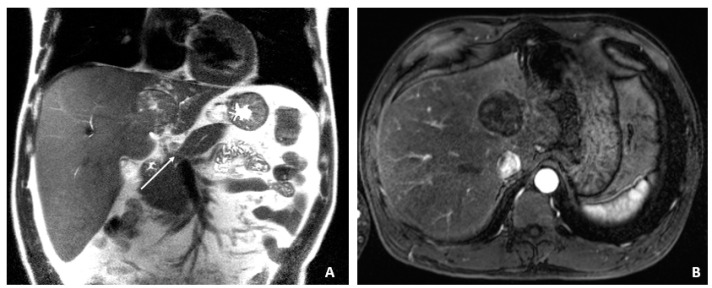
MRI at the time of diagnosis of HCC (December 2009). (**A**) The coronal T2-weighted image shows a mildly heterogeneous mass in the left hepatic lobe (arrow), corresponding to hepatocellular carcinoma. (**B**) The axial T1-weighted fat-suppressed arterial-phase postcontrast image shows faint foci of hyperenhancement within the lesion, characteristic of HCC.

**Figure 2:**
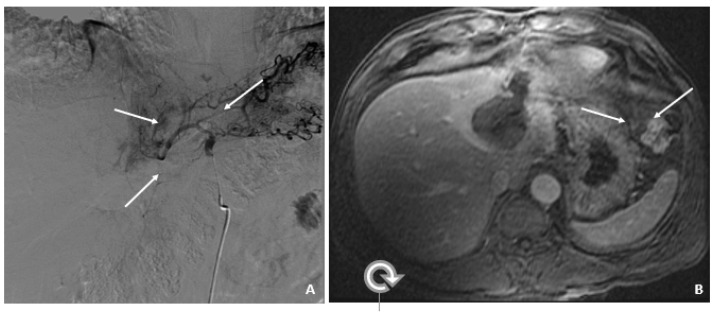
Transarterial Chemoembolization (December 2009). (**A**) The selective left hepatic artery injection shows a blush of contrast corresponding to the hypervascular tumor (arrows). (**B**) An axial T1-weighted fat-suppressed portal-phase postcontrast MRI image 4 weeks following TACE shows lack of enhancement in the treated lesion with a small adjacent focus of necrosis (arrows).

**Figure 3:**
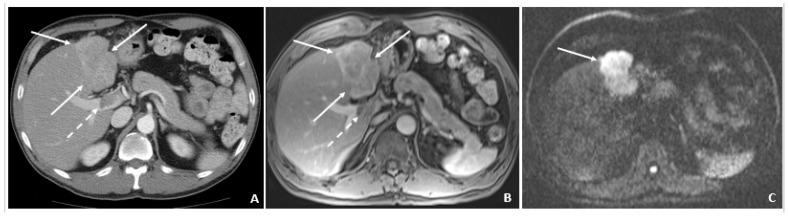
Recurrent HCC 2 Years Later (April 2011). (**A**) The contrast-enhanced CT image shows a hyperenhancing lesion in the left lobe (arrows) with adjacent tumor thrombus within the portal vein (dashed arrow). (**B**) The subsequent axial T1-weighted fat-suppressed postcontrast MRI image shows the lesion (arrows) to a slightly better advantage, along with the portal vein tumor thrombus (dashed arrow). (**C**) The diffusion-weighted image shows marked hyperintensity (arrow) within the left lobar mass, corresponding to diffusion restriction, typical of malignant tumors.

A 50-year-old male was diagnosed with CHB at age 40. With lamivudine treatment for 3 years, he achieved HbsAg seroconversion to anti-HBs and lamivudine was discontinued. Table 4 outlines his course. Five years later, on a follow up examination he was found to have HBV DNA 847 copies/mL, ALT 33 IU/L, and AFP 20.5 ng/mL. An MRI (December 2009) showed a 5 cm lesion consistent with HCC (Figure 1A,B). Repeat AFP was 17.8 ng/mL at time of HCC diagnosis, and AFP-L3% was 75.6. He underwent transarterial chemoembolization (TACE) with successful resolution (Figure 2A,B) and restarted lamivudine. The AFP remained at 3.0 ng/mL, and the MRI showed no evidence of HCC the following year. Two years after initial treatment, the AFP increased to 5.9 ng/mL with 57.5% L3. The MRI next month showed recurrent HCC at the treated site (Figure 3). The patient underwent laparoscopic radiofrequency ablation and therasphere treatment.

Subsequent %L3 AFPs remained persistently high with minimally elevated total AFP levels, which indicated recurrent/progressive HCC.

### 6.5. Case 5: A 58-Year-Old Male

A 58-year-old male was found to have CHB on a routine examination in May 2007. HBV DNA was 4.6 × 10^5^ copies/mL. He was started on telbivudine. The AFP was 3.2 ng/mL in May 2008 with AFP-L3% of 13.8. The MRI in July 2008 showed cirrhosis and no tumor. In August 2008, the AFP remained low at 3.2 ng/mL, but there was a rise in the AFP-L3% to 33.6. The AFP-L3% continued to rise to 69.7 by November 2008, while the absolute AFP value remained at 7.9 ng/mL. In March 2009, the AFP rose to 24.1 ng/mL, and the AFP-L3% at this time was continuing to rise to 87.7. At this time, the abdominal MRI showed a 2.4 × 2.0 cm HCC. He underwent cryoablation followed by radiofrequency tumor ablation and TACE (March and August 2009). These values are outlined in Table 5.

## 7. Discussion and Conclusions

We present this case series of five patients with HBV infection who have elevations in AFP at various points in their disease course. We retrospectively analyzed the %L3 of AFP in these samples to evaluate for the possibility of usefulness of routine L3% evaluation along with AFP screening in patients with CHB at risk for HCC.

Of the five patients, Case 1 had CHB with ALT flare and Case 2 had liver cirrhosis. Both had elevated AFP levels with a relatively low percentage of AFP-L3 (usually below 10%) in the setting of nonmalignant hepatocellular damage and concomitant liver cell regeneration. No signs of tumor recurrence were noted in these patients.

Cases 3, 4, and 5 demonstrate patients with levels of AFP and L3% varying at different points in their disease course including time of HCC diagnosis, resolution of HCC, and recurrence of HCC. They demonstrate high AFP with elevated L3% correlating with active HCC disease. Specifically for Case 5, we can see rising AFP-L3% (>10%) prior to a significant absolute increase in the value of AFP and prior to HCC being identified on imaging. This suggests the use of AFP-L3% being used as an early screening marker to detect small HCCs that are not yet detectable by AFP values alone or on imaging. We see in each of these cases, when HCC is present, regardless of the level of AFP alone, the L3% is greater than 10%, which demonstrates that L3% specifically may be highly sensitive for detection of initial disease as well as recurrence.

These cases also demonstrate the utility of AFP-L3% for monitoring for recurrence in patients who have had HCC treated. This is demonstrated nicely in Case 4, as the AFP value and AFP-L3% came down significantly after TACE treatment in concordance with imaging findings indicating resolution of HCC. This resolution was followed by an abrupt and significant rise in AFP-L3% (57.5% from 0.5%), which was associated with a relatively smaller and nonspecific rise in the absolute value of AFP (5.9 ng/mL from 2.5 ng/mL) with subsequent imaging findings consistent with recurrent HCC.

Overall, these cases demonstrate the utility of AFP-L3% in differentiating between various causes of AFP elevation including ALT flare, cirrhosis, and HCC, and the utility for early detection of HCC and recurrence after treatment. The usefulness of the combination of AFP, AFP-L3, and DCP for early detection of HCC is evident, and notably the GALAD scoring algorithm, which has been reported in the past [16,17,18,19,20]. In our similar study, we further noted that AFP-L3 is particularly useful in predicting the recurrence of HCC after tumor ablation [16]. Our current study re-confirmed the significance of AFP-L3 for early detection of recurrence in the presence of low AFP and long before the radiological confirmation.

This use of AFP-L3% in conjunction with standard imaging +/− AFP screening warrants further studies to demonstrate the utility in routine HCC screening. In a study done by Hann et al. in 2014, they noted that AFP-L3% was useful in patients with low levels of AFP (<20 ng/mL), and they saw that a significant AFP-L3% elevation could be detected over one year before the diagnosis of HCC as well as predicting recurrence of HCC [16]. These cases demonstrate the need for more studies to further support the use of AFP-L3% in early detection of HCC before a small lesion is able to be detected on imaging and before a significant rise in absolute AFP value is detected.

The use of AFP for routine HCC screening is controversial and is often regarded as a better tool for monitoring treatment response and recurrence rather than a tool for initial diagnosis. This is likely because AFP can be elevated for a number of non-malignant reasons, which may lead to unnecessary additional imaging. It is therefore possible that, by measuring the %L3 in addition to AFP, it can increase the diagnostic power of AFP by helping to distinguish an AFP elevation due to nonmalignant hepatic inflammation from HCC development and recurrence.

## Figures and Tables

**Figure 4:**
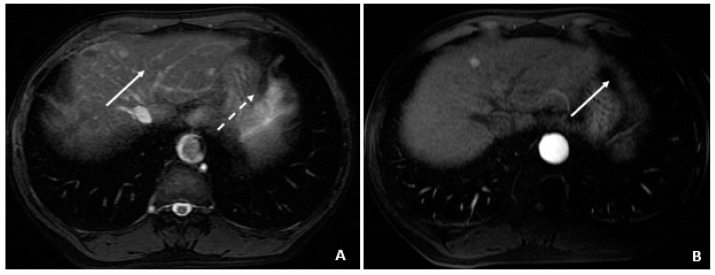
Initial MRI with normal AFP and elevated %L3 (July 2008). (**A**) The axial T2-weighted gradient echo image shows a small hyperintense lesion (arrow) in segment 4A. A second hyperintensity (dashed arrow) corresponds to a hemangioma. (**B**) The corresponding axial T1-weighted fat-suppressed arterial-phase postcontrast image shows hyperenhancement (arrow) in the lesion subsequently found to be a small HCC, although currently without associated features to clinch the diagnosis. Without washout, capsule appearance, or other typical HCC findings, the lesion is indeterminate.

**Figure 5:**
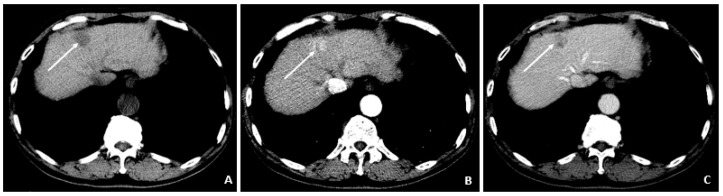
Triphasic CT at the time of HCC diagnosis (March 2009). (**A**) The precontrast image shows a hypodense lesion (arrow) at the site of the punctate hyperenhancing lesion shown in Figure 4. (**B**) The corresponding arterial-phase postcontrast image reveals hyperenhancement (arrow). (**C**) The portal-phase postcontrast image shows washout (arrow), and all features are typical of HCC and confirm the diagnosis in the setting of chronic HBV.

**Figure 6:**
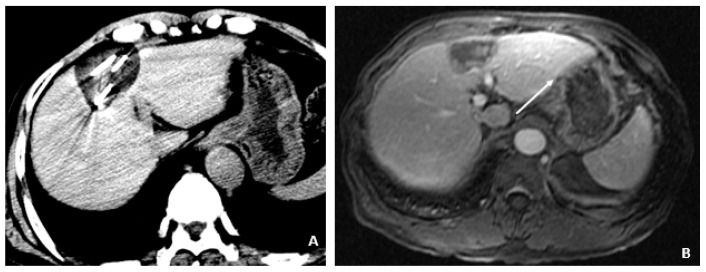
Cryoablation of the segment 4A HCC (April and May 2009). (**A**) The CT image through the region of the segment 4A HCC shows the cryoprobes and formation of the surrounding ice ball that accumulates as freezing cycles are applied to ablate the lesion. (**B**) A delayed postcontrast T1-weighted fat-suppressed image 1 month later shows little to no apparent enhancement in the ablated lesion (arrow).

**Figure 7:**
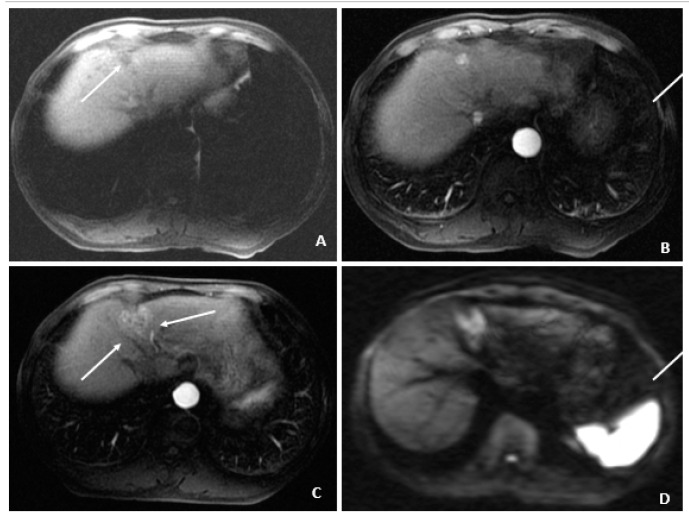
Recurrent HCC after cryoablation (August 2009 and July 2010). (**A**) At 3 months following cryoablation, the axial T1-weighted fat-suppressed precontrast image shows a small hypointense lesion (arrow) above and in proximity to the recently ablated HCC. (**B**) The corresponding arterial-phase postcontrast image shows hyperenhancement (arrow), but additional features (i.e., washout, capsule appearance, etc.) were not observed. (**C**) Months later, a follow-up MRI showed threshold growth of the hyperenhancing lesion (arrows). (**D**) The corresponding diffusion-weighted image shows diffusion restriction (arrows) and helps to confirm the diagnosis of HCC (as an ancillary feature); washout was also evident (not shown), confirming the diagnosis of HCC.

**Table 1:** Case 1. AFP, %L3, and ALT during each following year after initial elevation in AFP.

Date	AFP (ng/mL)	AFP-L3 (%)	ALT (IU/L)	Status
July 2008	1896	4.4	149	ALT flare, no HCC
June 2009	3	0.5	WNL	No ALT flare, no HCC
September 2010	3	0.5	WNL	No ALT flare, no HCC

**Table 2:** Case 2. AFP, L3%, and ALT at the time of cirrhosis diagnosis and after initiation of treatment.

Date	AFP (ng/mL)	AFP-L3 (%)	ALT (IU/L)	Status
June 2011	195.6	6.9	331	Cirrhosis
June 2011	315.7	8.9	336	Cirrhosis
June 2011	360.9	10.4	250	Cirrhosis
July 2011	332.8	11.5	150	Cirrhosis
September 2012	85.7	8.7	32	Cirrhosis

**Table 3:** Case 3. AFP, L3%, and ALT at time of HCC recurrence.

Date	AFP (ng/mL)	AFP-L3 (%)	ALT (IU/L)	Status
July 2004	8.4	74.2	49	HCC recurrence

**Table 4:** Case 4. AFP, L3%, ALT, and MRI findings at various times throughout treatment course.

Date	AFP (ng/mL)	AFP-L3 (%)	ALT (IU/L)	MRI	HCC Treatment
December 2009	17.8	75.6	33	5 cm HCC (Figure 1A,B)	TACE (Figure 2A,B)
March 2010	3.0	0.5	29	No evidence of HCC	
August 2010	2.5	0.5	25	No evidence of HCC	
March 2011	5.9	57.5	48		
April 2011				Recurrent HCC (Figure 3A–C)	Laparoscopic-RFA Therasphere Treatment
May 2011	14.5	48.8	59		
June 2011	11.3	53.6	550		
July 2011	18	53.8	69	Progression of HCC	TACE

**Table 5:** Case 5. AFP, L3%, ALT, and imaging findings at time of HCC screening, diagnosis, and at various treatment points.

Date	AFP (ng/mL)	AFP-L3 (%)	ALT (IU/mL)	MRI	HCC Treatment
May 2008	3.2	13.8	32	No tumor	
August 2008	3.2	33.6	20	July 2008 (Figure 4A,B) Indeterminate	
November 2008	7.9	69.7	30	No tumor visualized	
March 2009	24.1	87.7	17	2.4 × 2.0 HCC (Figure 5A–C)	
March 2009	16.6	88.8	23	HCC	
April 2009 May 2009					Cryoablation (Figure 6A,B)
August 2009 July 2010	6.7	74.7	20 (8/17/09)	new HCC (Figure 7A–D)	RFA, TACE

## Data Availability

Not applicable.
